# Strategies for mitigating inter-crystal scattering effects in positron emission tomography: a comprehensive review

**DOI:** 10.1007/s13534-024-00427-7

**Published:** 2024-09-17

**Authors:** Min Sun Lee, Hyeong Seok Shim, Jae Sung Lee

**Affiliations:** 1 Environmental Radioactivity Assessment Team, Nuclear Emergency & Environmental Protection Division, Korea Atomic Energy Research Institute, Daejeon, Republic of Korea; 2 Interdisciplinary Program in Bioengineering, Seoul National University, Seoul, Republic of Korea; 3 Integrated Major in Innovative Medical Science, Seoul National University Graduate School, Seoul, Republic of Korea; 4 Department of Nuclear Medicine, Seoul National University College of Medicine, 103 Daehak-ro, Jongno-gu, Seoul, 03080 Republic of Korea; 5 Brightonix Imaging Inc, Seoul, Republic of Korea

**Keywords:** Compton scattering, Inter-crystal scattering, Positron emission tomography, Triple coincidences

## Abstract

Inter-crystal scattering (ICS) events in Positron Emission Tomography (PET) present challenges affecting system sensitivity and image quality. Understanding the physics and factors influencing ICS occurrence is crucial for developing strategies to mitigate its impact. This review paper explores the physics behind ICS events and their occurrence within PET detectors. Various methodologies, including energy-based comparisons, Compton kinematics-based approaches, statistical methods, and Artificial Intelligence (AI) techniques, which have been proposed for identifying and recovering ICS events accurately are introduced. Energy-based methods offer simplicity by comparing energy depositions in crystals. Compton kinematics-based approaches utilize trajectory information for first interaction position estimation, yielding reasonably good results. Additionally, statistical approach and AI algorithms contribute by optimizing likelihood analysis and neural network models for improved positioning accuracy. Experimental validations and simulation studies highlight the potential of recovering ICS events and enhancing PET sensitivity and image quality. Especially, AI technologies offers a promising avenue for addressing ICS challenges and improving PET image accuracy and resolution. These methods offer promising solutions for overcoming the challenges posed by ICS events and enhancing the accuracy and resolution of PET imaging, ultimately improving diagnostic capabilities and patient outcomes. Further studies applying these approaches to real PET systems are needed to validate theoretical results and assess practical implementation feasibility.

## Introduction

Positron emission tomography (PET) is a diagnostic imaging tool with wide-ranging applications across various disease states [1–4]. This imaging technique operates by detecting pairs of 511 keV annihilation gamma-rays that arise from positron emission by radiopharmaceuticals within a patient's body. A ring-shaped PET system consisting of multiple PET detectors captures these coincidence events emitted in the opposite direction from the patient body and draws lines-of-response (LoR), which determines the location of annihilation interaction. The LoRs are then used to produce to a sinogram by using the systems’ essential spatial and angular information, and finally tomographic images are generated using various image reconstruction algorithms.

When a 511 keV gamma-ray enters a PET detector, it undergoes energy deposition through either photoelectric (PE) absorption or Compton scattering. In instances of Compton scattering, the resulting scattered photon transfer a portion of its energy across multiple crystal elements, giving rise to the phenomenon known as inter-crystal scattering (ICS). ICS events can lead to mis-positioning of gamma interactions within the crystal elements, introducing inaccuracies in the LoRs. Mis-positioned LoRs ultimately leads to degraded image quality [5].

In-depth understanding of the factors that causes ICS events within the PET detector is crucial to mitigate the challenges posed by ICS events and further improve PET image quality. This paper will cover the causes of ICS occurrence in PET and discuss its impact on the PET performances. Moreover, effective solutions for ICS event identification and recovery, which have been developed in an effort to enhance the accuracy and resolution of PET imaging will be introduced.

## ICS event in a PET detector

### Physics of ICS events

**Fig. 1:**
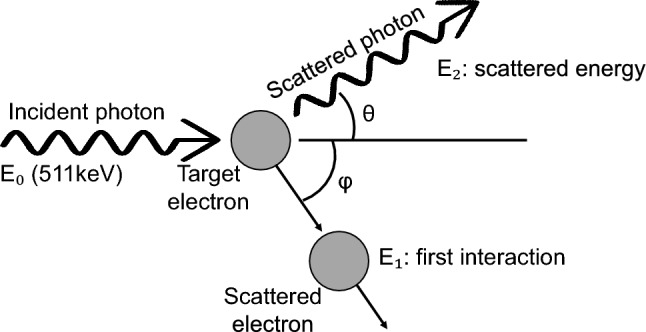
A schematic of Compton scattering event

When a 511 keV gamma-ray enters a PET detector, majority of 511 keV photons deposit their energy through either PE absorption or Compton scattering within the scintillation crystal. Pair production is a possible interaction between photons and matter; however, it does not occur at this energy level since it requires at least 1.022 MeV energy [6]. Compton scattering is one of major interactions between high-energy photons and an absorbing material. This interaction follows the principles of conservation of momentum and energy. After the collision between the incident photon and the target electron, the incident photon with energy *E*_0_ transfers a portion of its energy to the Compton recoil electron and the photon changes its direction and is scattered with the decreased energy *E*_2_ (Fig. 1). The energy difference (*E*_0_–*E*_2_) depends on the scattering angle ($\theta$), and this relationship is expressed by the Compton scattering equation as below:

1 $${E}_{2}= \frac{{E}_{0}}{1+ \frac{{E}_{0}}{{m}_{e}{c}^{2}}\left(1- \mathit{cos}\theta \right)}$$

where ${m}_{e}$ is electron rest mass and *c* is the speed of light.

**Fig. 2:**
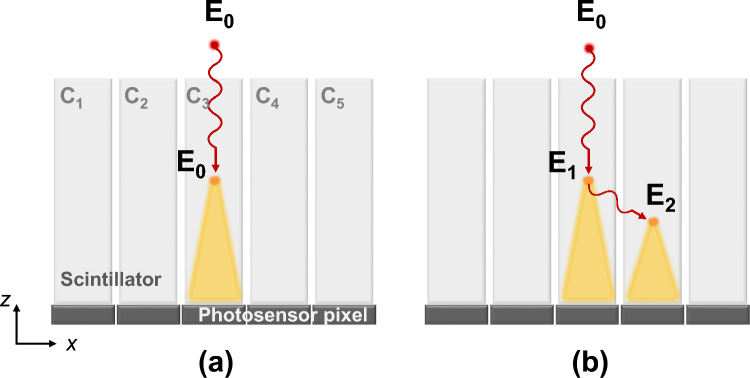
Event types in a typical pixelated detector where incident photon with energy *E*_0_ enters toward a single crystal. (**a**) Photoelectric absorption, where full energy *E*_0_ is deposited in a single crystal C_3_. (**b**) Inter-crystal scattering, where Compton scattered occurred only once within the detector. The energies *E*_1_ and *E*_2_ are deposited in two crystals of C_3_ and C_4_

The ejected Compton recoil electron undergoes scintillation process as it interacts with the crystal lattice. Meanwhile, if the scattered photon travels to adjacent crystal elements and deposit its energy, leading to dispersion of 511 keV gamma-ray energy across multiple crystal elements, we recognize it as inter-crystal scattering (ICS) event as shown in Fig. 2. In Fig. 2, we consider the case where Compton scattering occurs once within the pixelated detector. We assume that the energy of the Compton recoil electron (*E*_1_) and the scattered photon (*E*_2_) are fully absorbed by the crystal where the interaction takes place.

The probability of Compton scattering occurring at the specific scattering angle ($\theta$) is described by the Klein-Nishina formula as below:

2 $$\frac{{d{\upsigma }}}{{d{\Omega }}} = \frac{1}{2}r_e^2 \left( {\frac{{{\text{E}}_{2} }}{{{\text{E}}_{0} }}} \right)^2 \left( {\frac{{{\text{E}}_{0} }}{{{\text{E}}_{2} }} + \frac{{{\text{E}}_{2} }}{{{\text{E}}_{0} }} - (sin\theta )^2 } \right)$$

where $\frac{{\text{d}}\upsigma }{{\text{d}}\Omega }$ is the differential cross-section with respect to the solid angle $\Omega$ and ${r}_{e}$ is the classical electron radius.

**Fig. 3:**
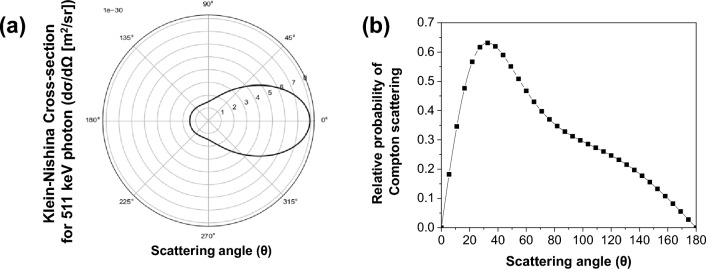
Angular distribution of scattered photon resulting from Compton scattering. (**a**) Klein-Nishina cross-section $\left( {\frac{{d{\varvec{\sigma }}}}{{d{\varvec{\Omega }}}}} \right)$ distribution of Compton scattering along scattering angle ($\theta$) into a unit solid angle (**b**) Relative probability of Compton scattering angles from 0 to 180 degrees. Maximum peak is observed at 35 degrees

The Klein–Nishina formula represents the cross-section of Compton scattering, indicating the probability of scatter (scattering) per unit solid angle as shown in Fig. 3. This formula quantifies the probability of a scattered photon emerging at a specific energy and angle, utilizing the angle of scatter and the energy divided by scatter as variables. As shown in Fig. 3b, forward scatter is more likely to occur, with the scattering angle smaller than 90 degrees. According to the Compton scattering equation, the distribution of energy resulting from scatter at a specific angle is determined, thereby assigning fixed values for the angle at which scatter occurs. Details will be provided in the following Sect. 4.1.

### Occurrence of ICS events

In a PET detector, the occurrence of ICS events is affected by the size and the composition of scintillation material used in the detector array, which determines the attenuation length of 511 keV gamma-ray within the detector array. Additionally, the size of individual scintillation crystal elements or pixels that comprise the detector array is a crucial factor in determining the occurrence of ICS. Detectors composed of smaller scintillation crystal elements are naturally more prone to experiencing ICS, and scintillation materials with lower density and stopping power may contribute to an increased occurrence of ICS phenomena.

#### Size of detector element

**Fig. 4:**
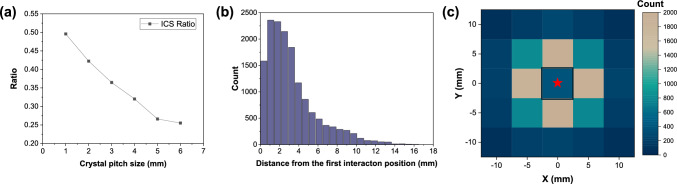
Monte Carlo simulation results of ICS occurrence ratio for a 25.4 × 25.4 × 20 mm^3^ LSO crystal block (**a**) with different crystal pitch size from 1 to 6 mm, and (**b**) the distribution of distance from the first interaction position to final energy deposition. (**c**) ICS occurrence distribution for 5 mm crystal pitch case, where the black box at the middle shows the first interaction position and red star shows the 511-keV gamma source position

We evaluated the ICS phenomenon through a GATE Monte Carlo simulation study, by irradiating an isotropic 511 keV gamma-ray point source at the center (0, 0, 0) mm of a 25.4 × 25.4 × 20 mm^3^ detector block for the widely used LSO crystal in PET. The LSO crystal pitch varied from 1 to 6 mm with 0.1 mm gap between crystals which filled with Teflon, and valid events that entered energy window of 400 and 600 keV was used. This energy window range was selected because it represents a widely accepted window for double coincidence measurements, which is general practice in many PET systems, particularly when dealing with varying detector resolutions. Additionally, a broader energy window can be applied when capturing triple coincidence events. The proportion of ICS events among valid events—defined as instances where a 511 keV gamma-ray is completely absorbed in the detector block and fall within the energy window— reaches up to 50% for 1 mm pitch crystals (Fig. 4a). This results in 75% probability that at least one 511 keV gamma-ray of coincidence event will undergo ICS, which is a considerable amount. As expected, with the larger crystal pitch size, the proportion of ICS events among valid events decreases. Once the size of the crystal pitch reaches 6 mm, the proportion of ICS events among valid events reached 25%. This indicates that PET system with high-resolution applications is more likely to suffer from the ICS effects [7]. The distance from the first interacted crystal to the final absorbed crystal in ICS events is shown in Fig. 4b. The distance between the first interaction position and the final interaction position was calculated numerically. The graph indicates that ICS can occur not only between adjacent crystals but also between crystals located more than 15 mm apart within the detector block. Figure 4c also shows relevant results where the center crystal is the first interaction position and scattering are occurred in neighboring crystals. Given that ICS constitutes a significant portion of detection events in PET measurements, particularly for 511 keV photons, which have larger cross-sections for Compton scattering in typical crystal materials than for PE, overcoming the performance degradation caused by ICS is crucial [8].

#### Composition of detector element

The occurrence of ICS is not only influenced by the size of the crystal element but also by the material properties of the crystal. Various crystals with different effective atomic numbers and densities were tested. Simulations involved irradiating 511 keV at the center of a 25.4 × 25.4 × 20 mm^3^ crystal block with different crystal material, including NaI(Tl), BGO, LSO:Ce, and LaBr_3_ with 3 mm crystal pitch size. As shown in Table 1, crystals with higher effective atomic number and density are likely have lower probability of ICS occurrence.

**Table 1:** Occurrence of ICS in an 8 × 8 array of 3 × 3 × 20 mm^3^ crystal array with different crystal materials

	NaI(Tl)	BGO	LSO:Ce	LaBr_3_:Ce
Density (g/cm^3^)	3.67	7.13	7.40	5.3
Effective atomic number	50	73	66	46
Photon yield (ph/keV)	38	8	30	61
Occurrence of ICS (%)	54.3	32.2	36.5	60.9

#### Detector readout configuration

Prior to recovering ICS events to their first interaction position, it is essential to identify whether events went through ICS or not. The ideal method to resolve ICS events in a detector is to utilize a 1-to-1 coupling of a crystal element and a photosensor pixel, such as silicon photomultiplier (SiPM), with individual photosensor channel readout. However, it is not always desirable to use 1-to-1 coupling of crystal element and SiPM channel. Especially, the reduced size of SiPM for high-resolution applications, leads to decreased detection efficiency [9]. Also, most of PET systems use high degree of SiPM channel multiplexing because increased number of output channels place a high load on the subsequent data acquisition system [10–12].

Conventional charge division circuits, such as Anger logic and discretized positioning circuit [13–15], provide averaged interaction position information as in the center-of-gravity (CoG), making it difficult to identify multiple event positions and energy values for ICS events. The row-and-column sum method [16, 17] may help to identify ICS events, but it requires many readout channels. Hence, various multiplexing technique, which allows for the identification of ICS events from multi-array photosensors, have been proposed [12]. Moreover, combining the multiplexing scheme with shallow or deep neural network-based energy estimation methods have been suggested to identify ICS events from highly multiplexed SiPM channels [18]. By combining SiPM signals that have been modulated through different high-pass filters to produce distinct pulse shapes, the authors reduced 16 row-and-column sum signals into 4. An artificial neural network was then utilized for signal restoration. The proposed method allows for accurate energy estimation and identification of ICS events in PET detectors with only 4 signal readout channels from 8 × 8 SiPM array. The use of digital SiPM technology in which SiPM is integrated with digital readout circuitry will make it even easier to achieve individual readout compared to analog SiPM readout configurations [19].

In a light-sharing detector design, where one photosensor pixel is coupled with more than one crystal, identifying the interacted crystals and their respective energy depositions is even more challenging. Unlike a 1-to-1 coupling detector, a light-sharing design usually demands the use of a two-dimensional flood map generated using multiplexing circuits. While PE events in each crystal manifest as peaks in the flood map, ICS events are broadly distributed among the crystal positions determined by energy-weighted signal amplitudes.

## Impact of ICS events on the PET system

**Fig. 5:**
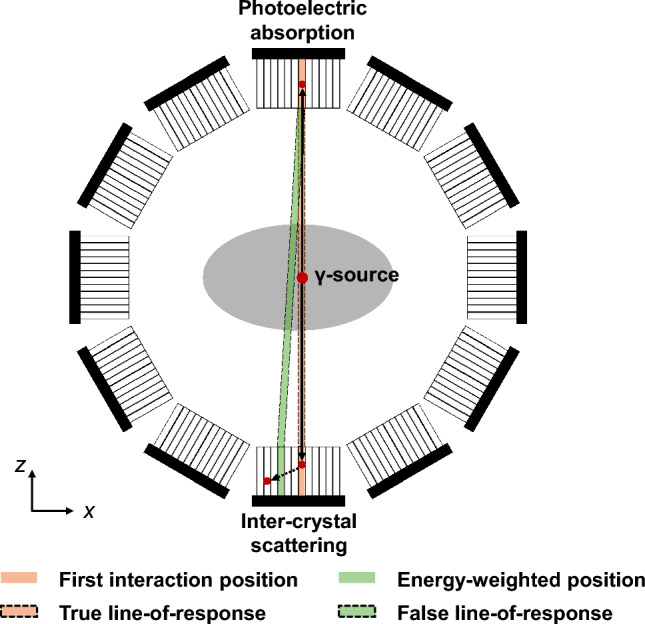
Inter-crystal scattering in a typical ring-shaped PET system

Accurate delineation of LoRs is crucial for obtaining high-quality PET images, including spatial resolution, signal-to-noise ratio (SNR), contrast, etc. As a standard procedure in PET, when two events fall within a specific time window, they are categorized as double coincidences. For detectors that do not have ICS resolving capability, ICS events cannot be distinguished from PE events. In a such detector configuration, multiple energy signals in a single detector are multiplexed into one energy signal (e.g. energy-weighted CoG), which may result in a wrong position (green crystal in Fig. 5). This results in misalignment of the LoRs, consequently degrading PET image quality.

For detectors that have ICS resolving capabilities, individual energy signal from each scattering interaction can be obtained. For a case when ICS event occurs in one detector, three events will fall within the time window, and they are categorized as triple coincidences. Triple coincidences resulting from ICS consist of two events from same detector and one event from another opposite detector. Here, we do not consider triple coincidence events that is caused by random events. For the triple coincidences, we can simply reject them to maintain the PET image quality, especially in terms of spatial resolution and image contrast [20, 21]. However, as discussed in the previous section, the occurrence of ICS events is significantly high, so excluding ICS events may not be desirable because it compromises the system sensitivity. Hence, it becomes crucial to recover ICS events into the first interaction position in order to accurately provide annihilation photon interaction positions and maintain system sensitivity at the same time.

As discussed in the previous section, the occurrence of ICS events varies depending on the composition, size, and configuration of PET detectors, as well as the geometry and measurement configurations (e.g. energy window, lowest energy threshold) of PET system itself. Thus, it is important to assess the impact of ICS on PET system performance, considering specific geometry and configuration of each PET system. This evaluation should consider aspects such as system sensitivity and image quality. Many research groups have investigated the effects of ICS on PET system performance based on their own system geometry [22–26].

## Methods for ICS event identification and recovery

Among valid events acquired within 511 keV energy window (e.g. 435–585 keV), ICS events can be distinguished from PE by using energy and position information of multiple interactions obtained from photosensors. It is important to note that the energy window range may vary depending on the energy resolution of the detector elements in the PET system. Among multiple interaction information, determining the first interaction position of an ICS event is the most important part. Various methodologies have been suggested for recovering ICS events into the first interaction position. In this section, different methods for ICS events recovery will be discussed. Additionally, we assume that object-scattered event is excluded by the energy window. Although distinguishing random events within the coincidence window is challenging, they can potentially be rejected by considering factors such as the distance between detector blocks.

### Energy comparison and Compton kinematics

#### Energy comparison

**Fig. 6:**
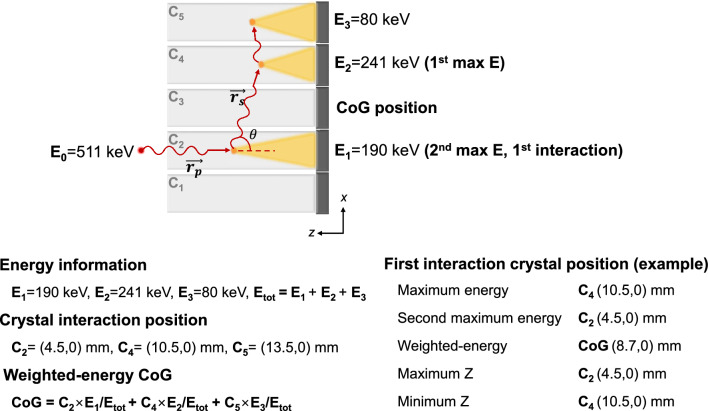
ICS recovery scheme based on energy comparison. Here, we assume a case when an incident gamma-ray with energy E_0_ undergoes two consecutive Compton scatterings at crystals C_2_ and C_4_, and finally absorbed at crystal C_5_, and energy is deposited at three crystal positions with energies E_1_, E_2_, and E_3_, respectively. C = (*x, y*) mm represents the position of each crystal center in the *x* and *y* directions

The simplest way to estimate the first interaction position of ICS events is to compare the energy absorbed by each crystal. Based on the acquired energy signal, the first interaction can be assigned to the crystal element where (1) maximum energy deposited, (2) second maximum energy deposited, and 3) CoG position from weighted-energy signals as shown in Fig. 6.

Comanor et al*.* [27] compared methods for recovering the first interaction position of ICS events based on energy information. They investigated and compared the maximum energy signal, second maximum signal, and joint algorithm for a 60-cm-diameter PET system with 2.5 cm axial field-of-view (FOV) consisted of 3 × 3 × 30 mm^3^ BGO crystals 1-to-1 coupled to 8 × 8 photosensor arrays. The joint algorithm designates the crystal as the first interaction based on the energy difference between two crystals where ICS occurred. When energy difference is larger than a specific threshold maximum signal is designated, and when energy difference is smaller than a specific threshold second maximum signal is designated. As a result, the second maximum signal and joint algorithm showed misidentification ratio of 12% which was better than that of maximum signal algorithm with 22% misidentification ratio.

Shao et al*.* [7] compared the accuracy of the first interaction positioning methods using maximum energy, maximum Z designating the first interaction position with the highest *z*-coordinated among the interaction positions, minimum Z, and weighted-energy scheme (Fig. 6). The maximum Z scheme exhibited highest ICS positioning accuracy for both 2 × 2 × 10 mm^3^ BGO and LSO, especially effective when gamma ray incident angle varied from 0 to 30 degrees. The minimum Z and maximum energy showed similar results, while the weighted energy scheme showed the worst performance.

**Fig. 7:**
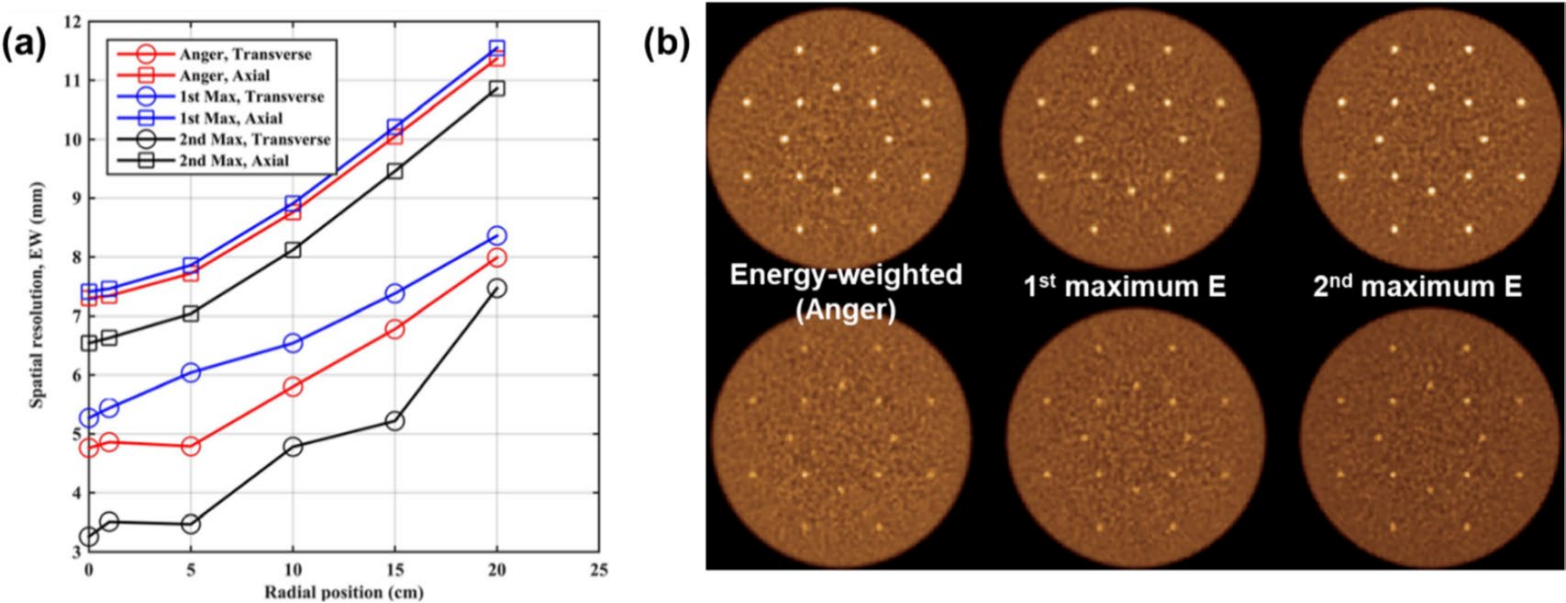
Comparison of energy comparison methods. (**a**) Reconstructed spatial resolution (equivalent width: total count in profile/peak count) of a point source in air plotted as a function of radial position for a 70 cm long whole-body scanner using 4 × 4 × 20 mm.^3^ LSO crystals. (**b**) Reconstructed images of the central transverse slices for the simulated lesion phantoms with 1 cm (top row) and 0.5 cm (bottom row) diameter lesions. Moving left to right in each row are images from Anger, 1st max crystal, and 2nd max crystal positioning algorithms. The water-filled cylinder was 35 cm in diameter and 70 cm long, and sphere uptake was 3:1 with respect to background. (From [28]; with permission)

Surti and Karp [28] investigated the positioning accuracy and analyzed the reconstructed image quality of using maximum energy deposition and second maximum energy, and compared them with weighted energy centroid positioning. The 1-to-1 detector coupling configuration was used while varying LSO crystal thickness from 1 to 3 cm and the crystal cross-section from 1 × 1 mm^2^ to 4 × 4 mm^2^. The positioning error, the distance from the true first interaction position to the recovered interaction position, was highest for the maximum energy in all cases, followed by weighted energy and second maximum energy. The second maximum energy deposition scheme demonstrated improved detector intrinsic spatial resolution and showed lower mean positioning error for crystal thicknesses of 1, 2, and 3 cm with all cross-section ranges. The system simulation was also conducted with a detector consisted of 4 × 4 × 20 mm^3^ LSO crystal and a system with an 85 cm ring diameter and a 70 cm axial FOV. The second maximum energy deposition scheme demonstrated improved system spatial resolution in both axial and transaxial directions and the contrast recovery coefficient values were enhanced by 30–40% compared to the maximum energy deposition scheme. (Fig. 7).

Energy comparison approach provides simple way to recover ICS events to the first interaction position, however, this approach requires 1-to-1 coupling of scintillation crystal to photosenstor pixel to obtain individual energy deposition at each crystal within the detector array. Overall, when comparing energy information for determining the first interaction position, the second-highest energy deposition method seems to offer better ICS recovery. This implies that a significant portion of Compton scattering occurs as forward scattering within the PET system, resulting in lower energy deposition at the initial interaction position (which also matches with Fig. 3b). However, it is not always accurate to assume that the second maximum energy deposition corresponds to the initial interaction position; the maximum energy deposited position can be the first interaction position when backscattering occurs. Hence, incorporating Compton kinematics in energy and position information of each crystal could help to improve positioning accuracy.

#### Compton kinematics-based method

Rafecas et al*.* [29] presented six recovery methods for ICS events in the MADPET-II, an LSO-APD-PET prototype PET system. They introduced Compton kinematics and Klein-Nishina equations to identify the crystal position where first interaction occurred. Six different methods were compared. (1) The averaging over LoRs (Av) scheme involves assigning a weight of 1/2 to each possible LoR and summing them. (2) The maximum energy scheme, as described in the previous section, draws LoRs based on the crystal with the highest signal. (3) The Compton kinematics scheme estimates the correct path by utilizing energy information and interaction position information through the Compton interaction formula (Eq. 1) to determine the scattering angle. (4) The modified Compton kinematics scheme considers errors in calculating the scattering angle. (5) The Klein-Nishina scheme assigns the trajectory with a higher probability between two trajectories using the Klein-Nishina formula. (6) The hybrid method is another modified Compton kinematics scheme, where the maximum energy scheme is used instead of Compton kinematics scheme under certain circumstances. Simulation study was conducted and reconstructed image analysis was performed.

The Compton kinematics scheme estimates the scattering trajectory by utilizing energy and interaction position information through Compton interaction formula (Eq. 1). Here, several assumptions are made: *E*_0_ equals 511 keV, the incident gamma-ray scatters only once and deposits the scattered radiation energy *E*_2_ in the second crystal, and the scattered radiation does not interact with non-sensitive detector materials, implying *E*_0_ = *E*_1_ + *E*_2_ = 511 keV. We can calculate the maximum energy transfer as *E*_1_ = 340.7 keV from Eq. 1, thus providing constraints for *E*_1_ and *E*_2_:

3 $${0\text{ keV}<E}_{1}\le 340.7\text{ keV and   }170.3\text{ keV}\le {E}_{2}<511\text{ keV}$$

Also, the scattering angle (*θ*) can be calculated in two different ways as described below, where $\overrightarrow{{r}_{p}}$ and $\overrightarrow{{r}_{s}}$ are the distance vectors corresponding to the primary and secondary trajectories, as indicated in Fig. 6. Ideally, the scattering angle calculated using measured energy information according to Eq. 4 and that calculated geometrically using the trajectories (Eq. 5) should yield the same value, but in real situations, they differ due to energy and position uncertainties.

4 $$\mathit{cos}{\theta }_{E}=2- \frac{{E}_{0}}{{E}_{0}-{E}_{1}}$$

5 $$\mathit{cos}{\theta }_{L}= \frac{\overrightarrow{{r}_{p}}\bullet \overrightarrow{{r}_{s}}}{\left|\overrightarrow{{r}_{p}}\right|\left|\overrightarrow{{r}_{s}}\right|}$$

The following explains how the first interaction position is finally determined in the Compton kinematics scheme, based on the measured energy signals from the photosensor, *E*_1_ and *E*_2_.

1. If *E*_1_ < 170.3 keV, *E*_2_ will be larger than 340.7 keV. In this case, the position with *E*_1_ is identified as the first interaction position.
2. If 170.3 keV < *E*_1_ and *E*_2_ < 340.7 keV, choose the one with the smaller ${K}_{L}$ value between the two possible scenarios (*E*_1_ or *E*_2_ corresponds to the first interaction). The ${K}_{L}$ represents the absolute difference between Eqs. 4 and 5, as calculated in the following equation:
  6 $${K}_{L}=\left|cos{\theta }_{E}-cos{\theta }_{L}\right|$$

The modified Compton kinematics scheme uses a modified ${K}_{L}$ value that incorporates uncertainties of energy and position information (${\sigma }_{L}^{2}$). Detailed error calculations can be found in this paper.

7 $${K}_{L}=\frac{\left|cos{\theta }_{E}-cos{\theta }_{L}\right|}{{\sigma }_{L}^{2}}$$

The Klein–Nishina scheme is based on Klein–Nishina formula described in Eq. 2. For two possible cases, probability of Compton scattering is calculated based on Klein-Nishina formula and LoR is chosen for the one that has higher probability. This approach allows for two possible implementations, depending on the method used to calculate $cos\theta$: either by energy considerations (KN–E, Eq. 4) or by geometrical analysis (KN–G, Eq. 5).

**Fig. 8:**
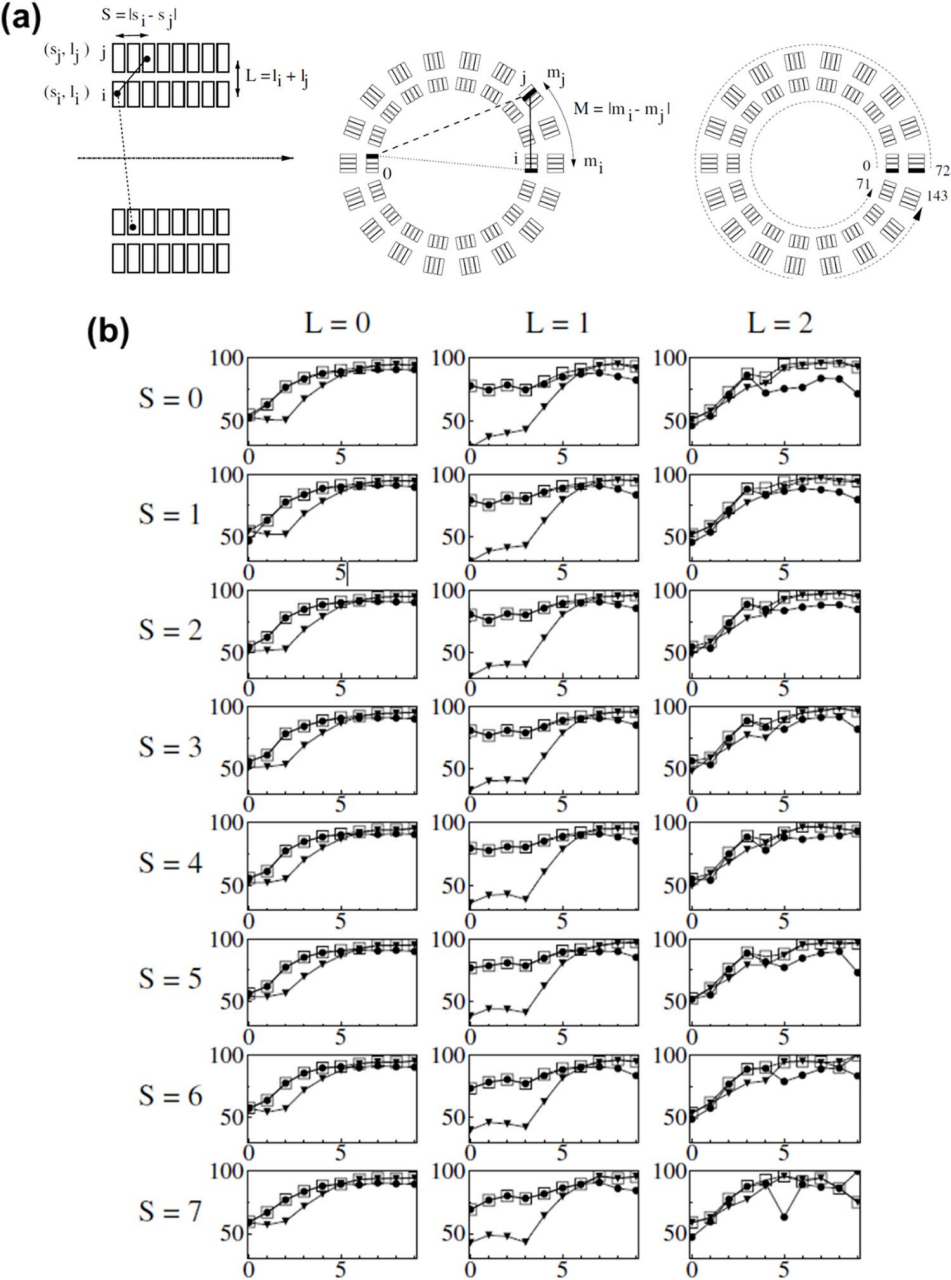
Comparison of ICS recovery methods for a dual layer, high resolution PET scanner. (**a**) Axial and frontal view of MADPET-II. The possible LoRs classified in (*M*, *S*, *L*) combinations with module differences (*M*_ij_), slice differences (*S*_ij_), and layer sums (*L*_ij_). Right figure shows the labelling of the channels in MADPET-II system. (**b**) Identification fraction (IF) for Maximum Energy (ME, ▼), Compton kinematics (Co, ●), and Hybrid scheme (HC, □) as a function of module difference M. For each case, the columns represent different values of L, and the rows represent values of S

Finally, the hybrid scheme is a modified Compton kinematics scheme by applying the maximum energy selection criterion under certain circumstances. For triple coincidences events, this method classifies two possible LoRs of ICS event into module differences (*M*_*ij*_), axial slice differences (S_*ij*_), and layer sums (*L*_*ij*_) (Fig. 8a). Depending on the (*M*_*ij*_*, S*_*ij*_*, L*_*ij*_) combinations, the identification fraction was pre-calculated using the Compton kinematics. For the combinations where the identification fraction was lower, maximum energy scheme was used instead of Compton kinematics.

The correct identification fraction of the first interaction position was highest with the value of 59.3% for the hybrid scheme, followed by 56.6% for Compton kinematics, 56.4% for modified Compton kinematics, 50.6% for maximum energy, and 49.9% for Klein-Nishina scheme as shown in Fig. 8b. The SNR of the reconstructed hot-rod PET images with the hybrid scheme was better than with other methods, except for the Av scheme. Contrast was also highest with the hybrid method.

Abbaszadeh et al*.* [26] also uses Compton kinematics to recover intra- and inter-crystal scatter in a CZT-based PET system. In 'cross-strip' CZT detectors, positioning is determined at the intersection of the responding anode strip and cathode strip. Consequently, in cases of intra- and inter-crystal scatter, more than one anode and cathode may respond, leading to the occurrence of four or more intersections. In such cases, energy-based methods are not applicable due to detector characteristics. Therefore, Compton kinematics are utilized to determine possible angles of trajectories. When applying Compton kinematics-based method with a low energy threshold of 10 keV, sensitivity improvements were observed with 1.43 times higher value and contrast-to-noise ratio (CNR) improvement from 5.81 to 12.53.

### Statistical approaches

Another approach to determining the first interaction position in ICS events involves employing optimization algorithms based on statistical or mathematical principles. Given that ICS events follows Compton kinematics and Klein–Nishina cross-section, Bayesian and Maximum Likelihood (ML) methods leverage probabilistic inference and likelihood maximization to estimate the first interaction position. These approaches use probabilistic models, thereby refining accuracy by integrating prior knowledge and updating probabilities that is based on Compton kinematics and Klein-Nishina formula.

When a 511 keV gamma-ray interacts at different positions after Compton scattering ${\varvec{x}}=[{x}_{1},{x}_{2},\dots ]$ with energy ${\varvec{E}}=[{E}_{1},{E}_{2},\dots ]$, observed detector response signal is represented by ${\varvec{S}}=[{s}_{1},\dots , {s}_{m}]$. We assume that each detector response follows Gaussian distribution with mean ($\mu ({\varvec{E}},{\varvec{x}})$) and the standard deviation ($\sigma$). When ICS event occurs at crystal position ${\varvec{x}}=[{x}_{1},{x}_{2}]$ with energy deposition ${\varvec{E}}=[{E}_{1},{E}_{2}]$, the probability that we observe detector response ${\varvec{S}}=\left[{S}_{1}, \dots {S}_{64}\right]$ from a 8 × 8 photosensor array can be described as $P\left({\varvec{E}},{\varvec{x}}|{\varvec{S}}\right)$ and the likelihood function is given in $\mathcal{L}({\varvec{E}},{\varvec{x}})$. For an estimate for maximizing $\mathcal{L}({\varvec{E}},{\varvec{x}})$ is given as Eq. 10 is known as ML estimate. Moreover, by incorporating a prior information or probability (e.g. in this case prior information will be Compton kinematics or Klein-Nishina cross-section), we can estimate parameters in a Bayesian framework. This is called Maximum a Posteriori (MAP) estimation as given in Eq. 11. Weighting parameter $\beta$ can be used to adjust relative importance of each component.

8 $${{P}}\left(E,x|S\right)=\prod_{{{i}}=1}^{{{m}}}\frac{1}{{{\sigma}}\sqrt{2{{\pi}}}}{e}{x}\mathbf{p}[-\frac{1}{2{{{\sigma}}}^{2}}{\left({{{s}}}_{{{i}}}-{{\mu}}\left(E,x\right)\right)}^{2}]$$

9 $$\mathcal{L}\left(E,x|S\right)={ln}\left({{P}}\left(E,x|S\right)\right)$$

10 $${{M}}{{L}}{{E}}\left(E,x\right)=\underset{{{E}},{ }{{x}}}{{argmax}}\mathcal{L}(E,x|S)$$

11 $${{M}}{{A}}{{P}}\left(E,x\right)=\underset{{{E}},{{x}}}{{argmax}}\mathcal{L}{\left(E,x|S\right)}^{1-{{\beta}}}{{{P}}}_{{{p}}{{r}}{{i}}{{o}}{{r}}}{\left(E,x\right)}^{{{\beta}}}$$

Pratx and Levin [30] presented a Bayesian method for reconstructing sequences of interactions in detectors to accurately identify the first interaction in multiple interaction events in their cross-strip CZT system. The MAP estimation based on Bayesian framework was used to optimize the sequence consistency while considering the trajectory total cross-section. The study compared the performance of MAP with other positioning methods–energy-weighted positioning and minimum distance positioning using simulations of CZT PET systems. MAP showed the first interaction positioning accuracy of 85.2% and significantly reduced the mispositioning of events compared to minimum distance method, leading to improved image quality, higher CNR, and better spatial resolution and thereby making it a promising technique for high-resolution PET imaging.

**Fig. 9:**
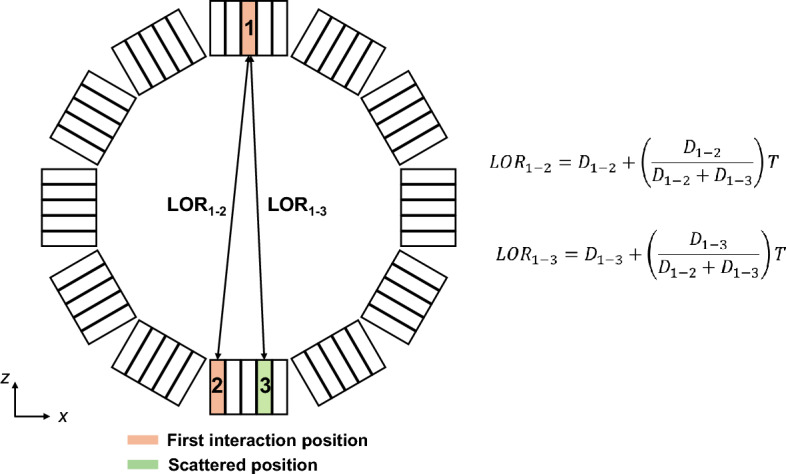
An ML approach without energy measurement. Example PET scanner with two possible LoRs for ICS events. The orange color crystal indicates the first interaction position and the green color crystal indicates the scattered crystal position

Lage et al. [31] presented an inter-detector scattering recovery method for recovering triple coincidences in PET without the need of additional energy resolution requirements. The authors distributed triple events (*T*) among their possible LoRs based on the relative proportions of double coincidences (*D*_1-2_ and *D*_1-3_) in those LoRs, representing a ML solution (Fig. 9). LoR information is presented in histogram format and described in count values; *D* and *T* components are also described as counts in LoR histogram format. This study adapted a real preclinical Argus PET/CT scanner to acquire and process triple coincidences and developed a normalization procedure specific to triple events. By including triple coincidences using their method, the peak noise-equivalent count (NEC) rates of the scanner increased by 26.6 and 32% for mouse- and rat-sized objects, respectively, leading to improved image quality with better signal-to-noise ratio while preserving spatial resolution and contrast. This ML-based inter-detector scattering recovery method also outperformed other approaches based on maximum energy, Compton kinematics, and machine learning in a simulation study [32] with the scanner geometry of SimPET, a MRI-compatible PET insert [33]. Furthermore, this approach has been extended to ICS and its impact on image quality of brain PET scanners has been evaluated [34].

Gross-Weege et al. [35] introduced a ML-based positioning algorithm for preclinical PET scanners. The ML algorithm compared expected light distributions with measured light distributions using Probability Density Functions (PDFs) generated from single-gamma-interaction models based on measured data. This approach led to a sensitivity gain of up to 19% over the CoG algorithm, without significantly impacting energy resolution or image quality. The study also demonstrated that the ML algorithm was less affected by missing channel information and could improve energy resolution and image quality by rejecting events that do not comply with the single-gamma-interaction model.

**Fig. 10:**
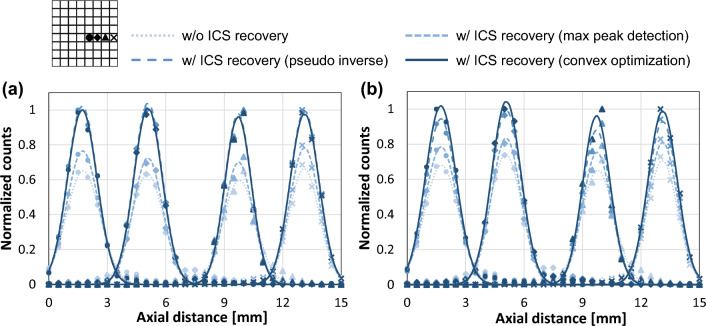
The impact of ICS recovery on detector performance. The intrinsic resolution profiles of four crystals in the one-to-one coupling detector with (**a**) the individual readout scheme and (**b**) the RC sum readout scheme. (From [36], with permission)

Lee et al. [36] treated the detector observation as a linear problem and offered two identification methods, pseudoinverse matrix calculation and convex constrained optimization and compared them with the maximum energy scheme. The study included simulation and experimental evaluations. The simulation study showed that the proposed convex optimization method yielded robust energy estimation and high ICS identification rates of 0.98 for the one-to-one coupling and individual readout with 8 × 8 3 × 3 × 20 mm^3^ crystal array and 0.76 for light-sharing detectors with 12 × 12 2.5 × 2.5 × 20 mm^3^ crystal, respectively. The experimental study showed a resolution improvement after recovering the identified ICS events into the first interaction position. The average intrinsic spatial resolutions improved by 1.5 times higher after applying ICS recovery using convex optimization method (Fig. 10). This proposed convex optimization method was applicable in typical pixelated light-sharing designs, even with a multiplexing readout scheme.

### Artificial intelligence

Artificial intelligence (AI) technology has gained significant attention and emerged as a predominant force across biomedical imaging and interpretation research [37–42]. Its applications extend beyond PET image processing, as AI technologies are actively employed to enhance the performance of PET detectors and readout technologies [3, 43, 44]. Hence many researcher groups have begun incorporating efforts to recover ICS events with various approaches using the AI technology.

Michaud et al. [45] pioneered the use of an AI-based method employing artificial neural network (ANN) to recover ICS events in PET systems. They utilized ANN on triple coincidence events to identify the correct LoR. The input of ANN consisted of position and energy information of triple coincidence data, which underwent preprocessing steps, such as normalization, energy sorting, and removal of symmetric events. A feed-forward multi-layer ANN structure was employed and trained using Monte Carlo simulation data of LabPET I [46] to acquire the best LoR recovery performance. The proposed ANN algorithm achieved a LoR recovery rate of 75 and a 55% sensitivity increase by including triple coincidences within 360–660 keV energy window. Despite some resolution degradation resulting from the inclusion of triple coincidence events rather than their rejection, the method's versatility across various conditions and pixelated detector systems renders it promising for PET imaging.

Recently, Wu et al. [47] explored the use of a feed-forward deep neural network (DNN) architecture for ICS recovery in 3D position sensitive detectors [48], which provide 3D position information: *x*, *y*, and *z* (depth-of-interaction, DOI). The authors utilized simulation data from a brain PET system with a 25 cm diameter and 5.4 cm length. This brain PET system consisted of detector modules arranged with 6 × 16 × 16 SiPM pixels, each 1-to-1 side-coupled with 3 × 3 × 3 mm^3^ LGSO crystals. The simulation assumed 12% energy resolution, an energy window of 400–600 keV, and a minimum detectable energy for any interaction of 10 keV. Here, a fully connected DNN was used with input comprising interaction position (*x, y, z*) and deposited energy information, capable of accommodating up to 10 scattering interactions per event. The first interaction position was used in training as label information. A softmax output layer produced a probability distribution for first interaction positions up to 10 possible positions; the position with the highest probability was then chosen. This DNN outperformed traditional algorithms such as maximum energy and lowest energy methods, achieving positioning accuracy ranging from 0.75 to 0.68 depending on the number of interactions per annihilation photons. Moreover, the DNN model showed better performance in image quality metrics such as contrast recovery (CR), CNR, and Modulation Transfer Function (MTF) values. The study suggests that DNN approach can enhance image quality and quantitation figures of merit in PET systems.

**Fig. 11:**
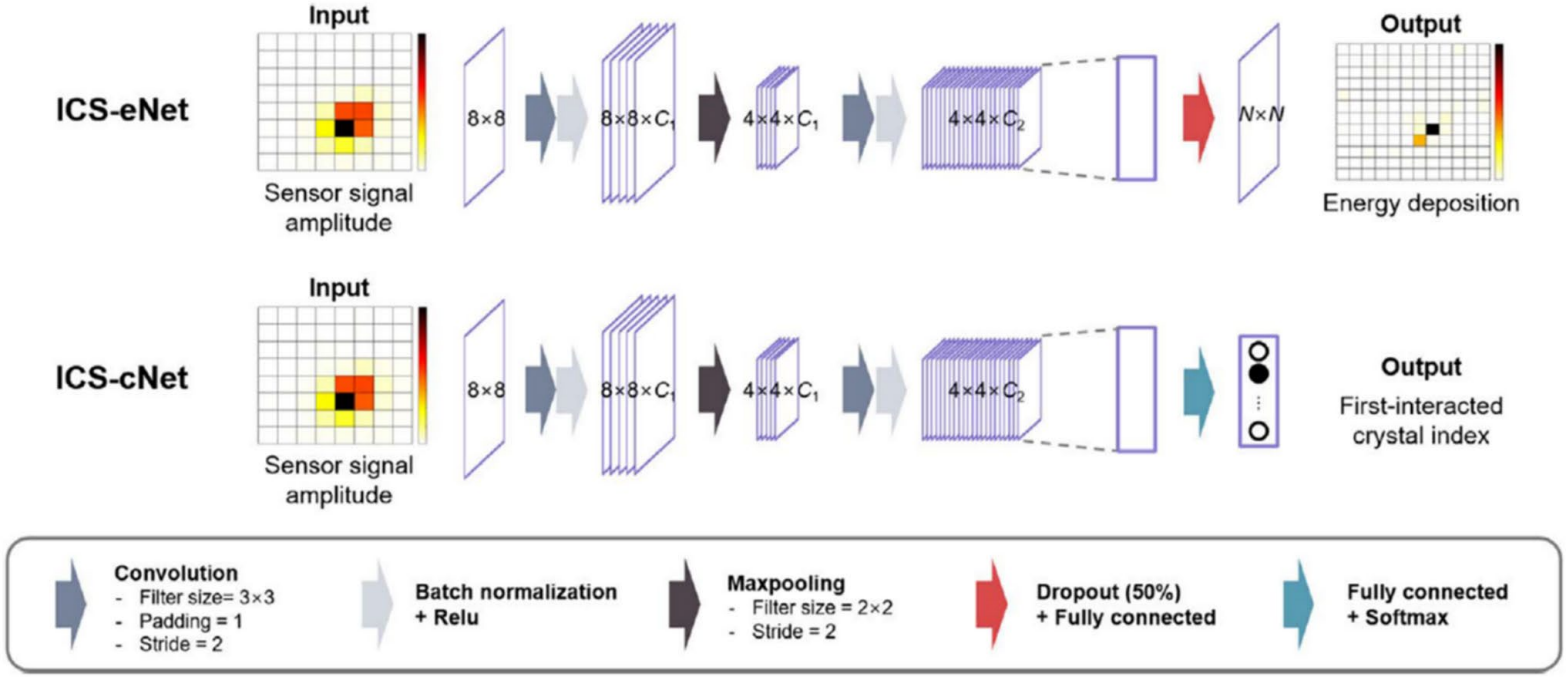
Structures of ICS-eNet and ICS-cNet. (From [49]; with permission)

A CNN (convolutional neural network)-based approach has been also proposed to recover ICS in conventional pixelated PET detectors with various crystal sizes [49, 50]. In these studies, the authors have developed two distinct network architectures: ICS-eNet, which estimates energies in individual crystals, and ICS-cNet, which directly determines the position of the first interaction crystal (Fig. 11). Simulations were conducted to generate input data with 8 × 8 to 21 × 21 LSO crystal arrays with length of 20 mm, along with the same photosensor array of 8 × 8 covering area of 25.8 × 25.8 mm^2^. In the simulation study, the CNN approach showed positioning accuracy of up to 90% for 1-to-1 coupled detector design, while the accuracy decreased as the degree of light sharing increased [49]. The authors also presented experimental validation using the proposed CNN network on the system level with 17 cm diameter virtual ring PET system. The CNN network achieved improvements of 11–46%, 33–50% in volume resolution in reconstructed image, and 47–64%, for 8 × 8, 12 × 12, and 21 × 21 arrays after ICS recovery, respectively [50].

**Fig. 12:**
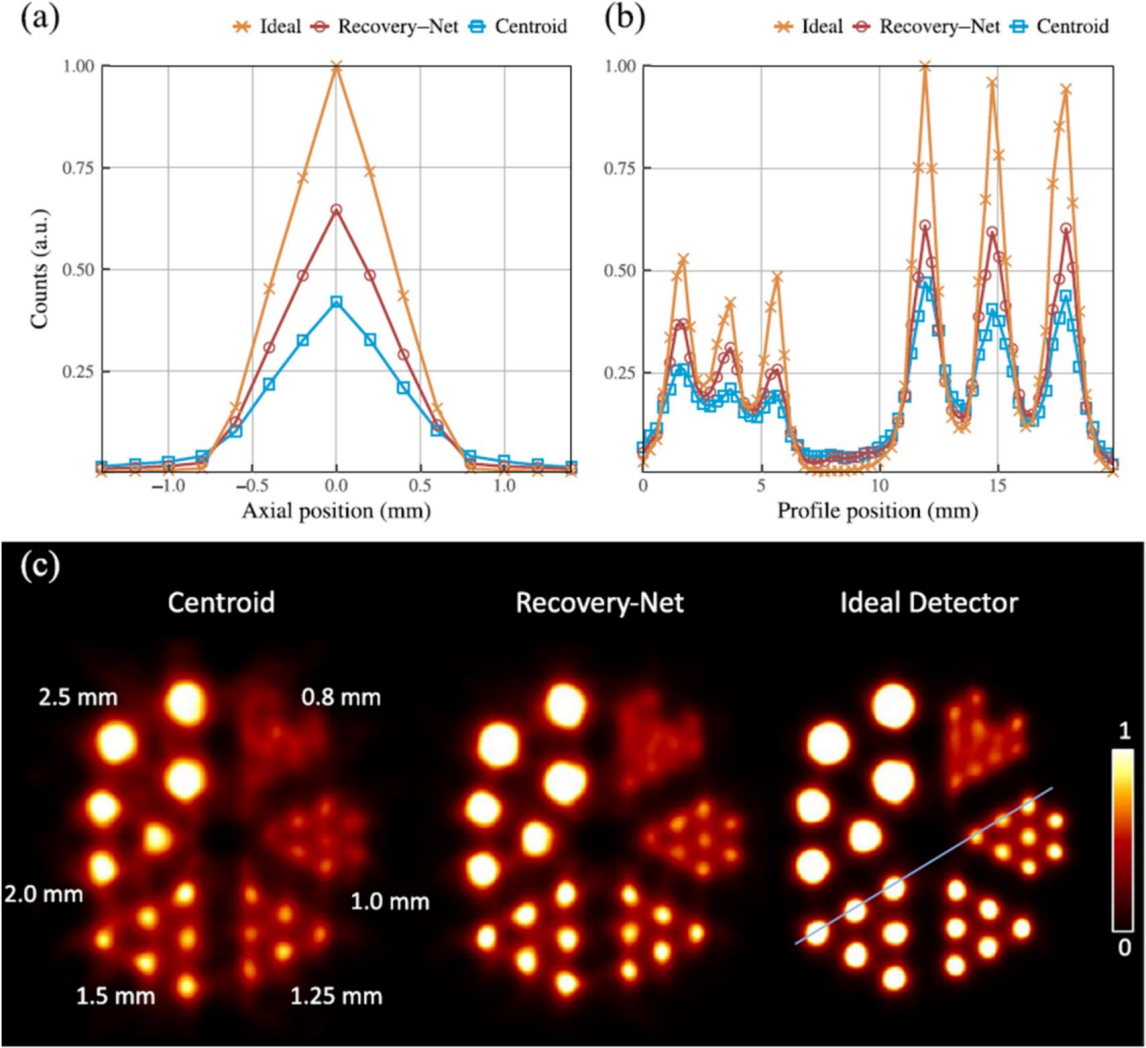
ICS recovery for a DOI detectors. (**a**) The count profiles were generated using three positioning approaches: centroid-based (no recovery), Recovery-Net, and an ideal detector (representing the upper limit of what's achievable). The displayed profile corresponds to a single pair of exactly opposed crystals, each positioned at the center of a Prism-PET detector module. (**b**) Line profiles were taken through 1.0 and 1.5 mm spots in the reconstructed image of an ultra-micro hot spot phantom. (**c**) The reconstructed images of the ultra-micro hot spot phantom were obtained under centroid-based, Recovery-Net, and ideal detector positioning schemes. (From [51]; with permission)

Petersen et al. [51] employed deep learning approach for their depth-encoding Prism-PET detector, achieving promising results for a light sharing detector. In this study, a simulated Prism-PET detector [52]—comprising a segmented light guide that enhances spatial and DOI resolution in a multi-crystal, single-ended readout— with 16 × 16 crystal array comprised of 1.4 × 1.4 × 20 mm^3^ LYSO coupled to 8 × 8 photosensor array was assumed. A Bayesian estimation method utilized a scatter absorption model as prior information and a detector response model as likelihood. For the machine learning approaches, they employed an ANN and a hybrid U-Net architecture (U-Net + autoencoder), which uses 8 × 8 sensor signal distribution as input. The output of the ANN was the energy and the DOI position (*z*) and the output of U-Net was the first interaction position and the DOI position (*z*), respectively. Due to the high degree of light sharing in the detector design, the Bayesian method yielded an estimation error of 20.5 keV in energy and 3.1 mm in DOI, while ANN achieved 26.2 keV in energy and 2.9 mm in DOI. By utilizing the hybrid U-Net architecture (Recovery-Net), 83% accuracy in crystal identification and 3.0 mm DOI estimation error was achieved, and image quality was significantly improved (Fig. 12). Furthermore, the results of several investigations [32, 47, 51, 53], in which the detectors with DOI information were utilized, suggest that incorporating DOI information can enhance the accuracy of ICS event position estimation.

## Discussion

ICS events pose significant challenges in PET imaging, affecting system sensitivity and the quality of reconstructed images including reconstructed image resolution and contrast. In this study, we aimed to provide a comprehensive review on the physics underlying ICS events occurring within PET detectors and the effective strategies developed to mitigate their impact on image quality.

Factors such as the size and composition of scintillation material influence the occurrence of ICS events, with smaller crystal elements and lower-density materials being more prone to ICS. For PET systems with low ICS occurrence, rejecting ICS events among valid events may be the optimal strategy to enhance image quality. Several simulation studies have shown that recovering ICS events into their true first interaction position can improve spatial resolution. However, in systems where the occurrence of ICS events is notably high, excluding them may not be desirable as it would ultimately degrade PET system sensitivity and image quality. Therefore, it is crucial to assess the impact of ICS on PET system performance, taking into account the specific geometry and configurations of each PET system. Consequently, recovering ICS events into their first interaction position becomes essential for accurately determining the annihilation photon interaction position and optimizing PET image quality.

A variety of methodologies have been proposed for recovering ICS events into their first interaction position, employing diverse approaches ranging from energy comparison and Compton kinematics to convex optimization-based algorithms and reconstruction-based system matrix modeling. Energy-based methods compare energy absorbed by each crystal, with the second-highest energy deposition method demonstrating better ICS recovery capabilities. Compton kinematics-based approaches utilize trajectory information to estimate the first interaction position, yielding promising results in improving PET image quality. Futhremore, convex optimization-based algorithms and reconstruction-based system matrix modeling leverage statistical and mathematical principles to estimate the first interaction position with high accuracy.

Moreover, AI technologies, including ANNs and CNNs, have emerged as promising tools for recovering ICS events in PET detectors. The neural network-based methods offer enhanced accuracy and image quality metrics compared to traditional algorithms, with several studies demonstrating significant improvements in positioning accuracy and spatial resolution. The integration of AI technologies into PET imaging represents a promising avenue for overcoming the challenges posed by ICS events and enhancing image quality. Table 2 summarizes pros and cons of main four approaches for ICS event recovery covered in this paper.

**Table 2:** Summary of ICS recovery approaches covered in this paper

	Pros	Cons
Energy comparison	Simple and fast; easy to implement in existing PET systems	Relatively lower accuracy in first interaction position estimation
Compton-kinematics based approach	More accurate identification than energy comparison; improved image quality	Sensitive to energy and position uncertainties
Statistical approach	Enhances accuracy by integrating prior knowledge; probabilistic models refine estimates	Requires significant computational resources; relies on accurate prior information
AI-based approach	Provides high accuracy and flexibility across various conditions; significant improvements in image quality and resolution	Requires extensive data and training; complex models can be difficult to interpret

Unfortunately, most studies to date have been based on the Monte Carlo simulation. While simulation studies have provided valuable insights, further research is essential to apply various ICS recovery algorithms to real PET systems and evaluate their usefulness. This practical implementation will validate theoretical results and assess the feasibility of real-world application. In addition, the development of metrics and evaluation methods for quantifying the impact of ICS on the system level and on PET image quality may be necessary. The occurrence of ICS varies depending on the system configuration. It is also important to note that the degradation of PET image quality is not solely caused by ICS; factors such as parallax error from DOI [54], positron range, and acolinearity also contribute to the quality degradation [34]. Therefore, isolating and evaluating the specific deterioration caused by ICS may provide us the valuable insights for future research.

Finally, an in-depth analysis on the effects of incorporating DOI information into the identification and recovery of ICS events is necessary. Utilizing DOI information can significantly enhance the accuracy of interaction position localization, thereby improving the precision of ICS event identification. This becomes particularly critical when the gamma source is positioned off-center within the PET system, as the 511 keV gamma-rays enter the detector element at oblique angles, potentially leading to increased positioning errors.

## Conclusion

In summary, ICS events in PET imaging pose challenges to image accuracy and quality. Understanding ICS physics is crucial for effective mitigation. Methods such as energy-based, Compton kinematics-based, convex optimization, and AI approaches aim to recover ICS events, promising improved imaging accuracy and resolution, with potential for enhancing diagnostic capabilities and patient outcomes.

## References

[1] Pomper MG, Lee JS. Small animal imaging in drug development. Curr Pharm Des. 2005;11:3247–72. 16250853 10.2174/138161205774424681

[2] Rahmim A, Zaidi H. PET versus SPECT: strengths, limitations and challenges. Nucl Med Commun. 2008;29:193–207. 18349789 10.1097/MNM.0b013e3282f3a515

[3] Lee JS, Lee MS. Advancements in positron emission tomography detectors: from silicon photomultiplier technology to artificial intelligence applications. PET Clin. 2024;19:1–24. 37802675 10.1016/j.cpet.2023.06.003

[4] Dhawan V, Niethammer MH, Lesser ML, et al. Prospective F-18 FDOPA PET imaging study in human PD. Nucl Med Mol Imaging. 2022;56:147–57. 35607632 10.1007/s13139-022-00748-4 PMC9123108

[5] Germano G, Hoffman EJ. A study of data loss and mispositioning due to pileup in 2-D detectors in PET. IEEE Trans Nucl Sci. 1990;37:671–5.

[6] Knoll GF. Radiation detection and measurement: John Wiley & Sons (2010).

[7] Yiping S, Cherry SR, Siegel S, Silverman RW. A study of inter-crystal scatter in small scintillator arrays designed for high resolution PET imaging. IEEE Trans Nucl Sci. 1996;43:1938–44.

[8] Wang Z, Dujardin C, Freeman MS, et al. Needs, trends, and advances in scintillators for radiographic imaging and tomography. IEEE Trans Nucl Sci. 2023;80:1244–80.

[9] Acerbi F, Paternoster G, Capasso M, et al. Silicon photomultipliers: technology optimizations for ultraviolet, visible and near-infrared range. Instruments. 2019;3:15.

[10] Ko GB, Yoon HS, Kim KY, et al. Simultaneous multiparametric PET/MRI with silicon photomultiplier PET and ultra-high-field MRI for small-animal imaging. J Nucl Med. 2016;57:1309–15. 27081173 10.2967/jnumed.115.170019

[11] Levin CS, Zaidi H. Current trends in preclinical PET system design. PET Clin. 2007;2:125–60. 27157870 10.1016/j.cpet.2007.12.001

[12] Park H, Yi M, Lee JS. Silicon photomultiplier signal readout and multiplexing techniques for positron emission tomography: a review. Biomed Eng Lett. 2022;12:263–83. 35892029 10.1007/s13534-022-00234-y PMC9308856

[13] Siegel S, Silverman RW, Yiping S, Cherry SR. Simple charge division readouts for imaging scintillator arrays using a multi-channel PMT. IEEE Trans Nucl Sci. 1996;43:1634–41.

[14] Park H, Ko GB, Lee JS. Hybrid charge division multiplexing method for silicon photomultiplier based PET detectors. Phys Med Biol. 2017;62:4390–405. 28368851 10.1088/1361-6560/aa6aea

[15] Goertzen AL, Zhang X, McClarty MM, et al. Design and performance of a resistor multiplexing readout circuit for a SiPM detector. IEEE Trans Nucl Sci. 2013;60:1541–9.

[16] Popov V, Majewski S, Welch BL. A novel readout concept for multianode photomultiplier tubes with pad matrix anode layout. Nucl Instrum Methods Phys Res A. 2006;567:319–22.

[17] Kwon SI, Lee JS. Signal encoding method for a time-of-flight PET detector using a silicon photomultiplier array. Nucl Instrum Methods Phys Res A. 2014;761:39–45.

[18] Shim H, Bae S, Lee S, Lee J. Inter-crystal scattering event identification using a novel silicon photomultiplier signal multiplexing method. Phys Med Biol. 2023;68: 115008. 10.1088/1361-6560/acd163 37116513

[19] Pratte J-F, Nolet F, Parent S, et al. 3D photon-to-digital converter for radiation instrumentation: motivation and future works. Sensors. 2021;21:598. 33467016 10.3390/s21020598 PMC7830581

[20] Gu Z, Taschereau R, Vu NT, Prout DL, Lee J, Chatziioannou AF. Performance evaluation of HiPET, a high sensitivity and high resolution preclinical PET tomograph. Phys Med Biol. 2020;65: 045009. 31935693 10.1088/1361-6560/ab6b44

[21] Kang HG, Tashima H, Wakizaka H, et al. Submillimeter-resolution PET for high-sensitivity mouse brain imaging. J Nucl Med. 2023;64:978–85. 36581375 10.2967/jnumed.122.264433 PMC10241014

[22] Gillam JE, Solevi P, Oliver JF, et al. Sensitivity recovery for the AX-PET prototype using inter-crystal scattering events. Phys Med Biol. 2014;59:4065. 24988897 10.1088/0031-9155/59/15/4065

[23] Hsu DFC, Freese DL, Innes DR, Levin CS. Intercrystal scatter studies for a 1 mm(3) resolution clinical PET system prototype. Phys Med Biol. 2019;64: 095024. 30893659 10.1088/1361-6560/ab115b

[24] Zhang C, Sang Z, Wang X, Zhang X, Yang Y. The effects of inter-crystal scattering events on the performance of PET detectors. Phys Med Biol. 2019;64: 205004. 31530747 10.1088/1361-6560/ab44f4

[25] Saaidi R, Rodríguez-Villafuerte M, Alva-Sánchez H, Martínez-Dávalos A. Crystal scatter effects in a large-area dual-panel Positron Emission Mammography system. PLoS ONE. 2024;19: e0297829. 38427663 10.1371/journal.pone.0297829 PMC10906883

[26] Abbaszadeh S, Chinn G, Levin CS. Positioning true coincidences that undergo inter-and intra-crystal scatter for a sub-mm resolution cadmium zinc telluride-based PET system. Phys Med Biol. 2018;63: 025012. 29131809 10.1088/1361-6560/aa9a2b PMC5785233

[27] Comanor K, Virador P, Moses W. Algorithms to identify detector Compton scatter in PET modules. IEEE Trans Nucl Sci. 1996;43:2213–8.

[28] Surti S, Karp JS. Impact of event positioning algorithm on performance of a whole-body PET scanner using one-to-one coupled detectors. Phys Med Biol. 2018;63: 055008. 29411709 10.1088/1361-6560/aaad76 PMC5876041

[29] Rafecas M, Böning G, Pichler B, Lorenz E, Schwaiger M, Ziegler S. Inter-crystal scatter in a dual layer, high resolution LSO-APD positron emission tomograph. Phys Med Biol. 2003;48:821–48. 12701889 10.1088/0031-9155/48/7/302

[30] Pratx G, Levin CS. Bayesian reconstruction of photon interaction sequences for high-resolution PET detectors. Phys Med Biol. 2009;54:5073–94. 19652293 10.1088/0031-9155/54/17/001 PMC3719884

[31] Lage E, Parot V, Moore SC, et al. Recovery and normalization of triple coincidences in PET. Med Phys. 2015;42:1398–410. 25735294 10.1118/1.4908226

[32] Lee S, Lee MS, Kim KY, Lee JS. Systematic study on factors influencing the performance of interdetector scatter recovery in small-animal PET. Med Phys. 2018. 10.1002/mp.13020. 10.1002/mp.13020 29851131

[33] Son J-W, Kim KY, Park JY, et al. SimPET: a preclinical PET insert for simultaneous PET/MR imaging. Mol Imaging Biol. 2020;22:1208–17. 32285357 10.1007/s11307-020-01491-y

[34] Lee S, Kim KY, Lee MS, Lee JS. Recovery of inter-detector and inter-crystal scattering in brain PET based on LSO and GAGG crystals. Phys Med Biol. 2020;65: 195005. 32575086 10.1088/1361-6560/ab9f5c

[35] Gross-Weege N, Schug D, Hallen P, Schulz V. Maximum likelihood positioning algorithm for high-resolution PET scanners. Med Phys. 2016;43:3049–61. 27277052 10.1118/1.4950719

[36] Lee MS, Kang SK, Lee JS. Novel inter-crystal scattering event identification method for PET detectors. Phys Med Biol. 2018;63: 115015. 29658493 10.1088/1361-6560/aabe3a

[37] Reader AJ, Corda G, Mehranian A, da Costa-Luis C, Ellis S, Schnabel JA. Deep learning for PET image reconstruction. IEEE Trans Radiat Plasma Med Sci. 2020;5:1–25. 10.1109/TRPMS.2020.3004408 PMC7610859 34056150

[38] Lee MS, Hwang D, Kim JH, Lee JS. Deep-dose: a voxel dose estimation method using deep convolutional neural network for personalized internal dosimetry. Sci Rep. 2019;9:10308. 31311963 10.1038/s41598-019-46620-y PMC6635490

[39] Lee JS. A review of deep-learning-based approaches for attenuation correction in positron emission tomography. IEEE Trans Radiat Plasma Med Sci. 2021;5:160–84.

[40] Rajendran P, Sharma A, Pramanik M. Photoacoustic imaging aided with deep learning: a review. Biomed Eng Lett. 2022;12:155–73. 35529338 10.1007/s13534-021-00210-y PMC9046497

[41] Rao D, Prakashini K, Singh R, Vijayananda J. Automated segmentation of the larynx on computed tomography images: a review. Biomed Eng Lett. 2022;12:175–83. 35529346 10.1007/s13534-022-00221-3 PMC9046475

[42] Alves VM, dos Santos CJ, Gama J. Classification of pulmonary nodules in [18F]FDG PET/CT images with a 3D convolutional neural network. Nucl Med Mol Imaging. 2024;58:9–24. 38261899 10.1007/s13139-023-00821-6 PMC10796312

[43] Berg E, Cherry SR. Using convolutional neural networks to estimate time-of-flight from PET detector waveforms. Phys Med Biol. 2018;63:02LT01. 10.1088/1361-6560/aa9dc5 PMC5784837 29182151

[44] Ullah MN, Levin CS. Application of artificial intelligence in PET instrumentation. PET Clin. 2022;17:175–82. 34809865 10.1016/j.cpet.2021.09.011

[45] Michaud JB, Tétrault MA, Beaudoin JF, et al. Sensitivity increase through a neural network method for LOR recovery of ICS triple coincidences in high-resolution pixelated- detectors PET scanners. IEEE Trans Nucl Sci. 2015;62:82–94.

[46] Bergeron M, Cadorette J, Beaudoin JF, et al. Performance evaluation of the LabPET APD-based digital PET scanner. IEEE Trans Nucl Sci. 2009;56:10–6.

[47] Wu C, Lee MS, Levin CS. Neural network-based inter-crystal scatter event positioning in a PET system design based on 3D position sensitive detectors. Conf Rec IEEE Nucl Sci Symp Med Imaging Conf. 2020;1:1–3.

[48] Cates JW, Levin CS. Evaluation of a clinical TOF-PET detector design that achieves ⩽100 ps coincidence time resolution. Phys Med Biol. 2018;63: 115011. 29762136 10.1088/1361-6560/aac504 PMC6016378

[49] Lee S, Lee JS. Inter-crystal scattering recovery of light-sharing PET detectors using convolutional neural networks. Phys Med Biol. 2021;66: 185004. 10.1088/1361-6560/ac215d 34438380

[50] Lee S, Lee JS. Experimental evaluation of convolutional neural network-based inter-crystal scattering recovery for high-resolution PET detectors. Phys Med Biol. 2023;68: 095017. 10.1088/1361-6560/accacb 37019126

[51] Petersen E, LaBella A, Li Y, Wang Z, Goldan AH. Resolving inter-crystal scatter in a light-sharing depth-encoding PET detector. Phys Med Biol. 2024. 10.1088/1361-6560/ad19f1. 10.1088/1361-6560/ad19f1 PMC11495245 38169459

[52] LaBella A, Cao X, Petersen E, et al. High-resolution depth-encoding PET detector module with prismatoid light-guide array. J Nucl Med. 2020;61:1528–33. 32111684 10.2967/jnumed.119.239343 PMC7539654

[53] Gu Z, Prout DL, Silverman RW, Herman H, Dooraghi A, Chatziioannou AF. A detector with crystal scatter identification capability for high sensitivity and high spatial resolution PET imaging. IEEE Trans Nucl Sci. 2015;62:740–7. 26478600 10.1109/TNS.2015.2408333 PMC4608445

[54] Ito M, Hong SJ, Lee JS. Positron emission tomography (PET) detectors with depth-of-interaction (DOI) capability. Biomed Eng Lett. 2011;1:70–81.

